# Extraction of Oil from Amazonian *Attalea tessmannii* Kernels: Kinetics Modeling, Diffusivity Analyses, and Physicochemical Characterization

**DOI:** 10.1021/acsomega.5c03451

**Published:** 2025-06-20

**Authors:** Sheraz Ahmad, Alice Neri da Silva Sousa, Viviane de Carvalho Arabidian, Keiti Roseani Mendes Pereira, Ricardo Scherer Pohndorf, Anelise Christ-Ribeiro, Isaac dos Santos Nunes, Débora Pez Jaeschke, Nauro da Silveira Junior, Luiz Antonio de Almeida Pinto, Tito Roberto Sant’Anna Cadaval Junior

**Affiliations:** † School of Chemistry and Food, 67820 Federal University of Rio Grande (FURG), Itália Avenue, km 8, Rio Grande, Rio Grande do Sul 96203900, Brazil; ‡ School of Exact and Natural Sciences, 37872 Federal University of Acre (UFAC), BR-364, km 4, Rio Branco, Acre 69920-900, Brazil; § Technology Development Center (CDTec), 37902 Federal University of Pelotas (UFPel), Campus Universitário, s/n, Capão do Leão, Rio Grande do Sul 96010-610, Brazil

## Abstract

The Amazon rainforest, recognized for its biodiversity, is an important source of timber and nontimber products that support the livelihoods of traditional communities. Among these resources, palm fruits are especially important because of their economic and ecological value. This study investigates *Attalea tessmannii*, an underexplored palm species, focusing on the chemical composition of its kernel, lipid extraction (kinetics and diffusivity analysis), and oil characterization. The kernel exhibited a high lipid content of 64.19%. Lipid extraction using hexane reached maximum yield at 60 °C after 180 min. Among the kinetic models tested, the Brimberg model showed the best fit, with an activation energy of 27.5 kJ mol^–1^. The diffusion coefficient ranged from 1.8 × 10^–11^ to 5.8 × 10^–11^ m^2^/s (25–60 °C). The oil was rich in short-chain fatty acids, mainly lauric acid (∼50%). The physicochemical parameters of *A. tessmannii* kernel oil indicated its potential for use in food, pharmaceutical, and biodiesel applications.

## Graphical abstract

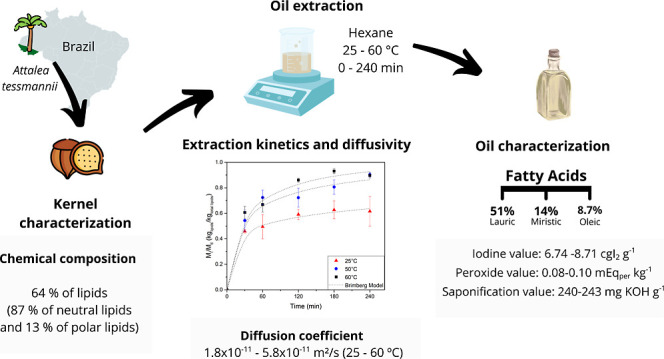

## 1 Introduction

The Amazon rainforest has great biodiversity and provides a variety of timber and nontimber products that support the income and survival of traditional communities. Nontimber forest products include various materials, such as fruits, nuts, herbs, and resins, that can be applied in different industrial sectors. Numerous of these materials remain underexplored and their sustainable exploitation has the potential to enhance the local economy through the production of chemicals and fuels, contributing to the conservation of the forest.^1,2^

Among the nontimber products, the fruits of palm trees (family *Arecaceae*) play a significant role in the forest ecosystem.^3^ The palm tree *Attalea tessmannii* is an underexplored vegetable that yields a fruit popularly referred to as “*cocão*”. This fruit is composed of epicarp, mesocarp, endocarp, and kernel. The kernel stands out as the most valuable part of the fruit due to its high oil content. It is commonly consumed raw or processed into a type of flour that is mixed with spices. Additionally, local cooperatives utilize cold pressing to extract the kernel oil, which is then used as an ingredient in regional cuisines. Furthermore, the remaining cake from the oil extraction is used to produce coal.^4^ However, these products are primarily consumed by local communities, and there is a lack of information in the literature regarding the composition of *A. tessmannii* kernels.

Other palm tree fruits from the same region, such as *babassu* (*Attalea speciosa*) and *buriti* (*Mauritia flexuosa*), are known for their high oil content, presenting mostly lauric, myristic, oleic, and palmitic fatty acids.^5,6^ These oils present high physical and chemical stability, along with emollient properties and bioactive compounds, being mostly used in the food and cosmetic industry as well as for biodiesel production.^7,8^ Hence, the kernel from *A. tessmannii* presents potential for the development of new products for use in food, pharmaceutical, and energy sectors. However, similar investigations on *A. tessmannii* are still missing. This gap points out the novelty of the present study and its contribution to a better understanding of the potential applications of this underexplored Amazonian palm, supporting the sustainable use of Amazonian biodiversity, and offering economic opportunities for traditional communities. Therefore, the central hypothesis of this study is that *A. tessmannii* kernels are a promising source of oil with favorable characteristics for use in food, cosmetic, or biofuel sectors.

The extraction of vegetable oils, such as those from palm kernels, typically involves the use of organic solvents like hexane. This solvent is often used because of its operational simplicity, effectiveness, controllability, and ease of recovery.^9^ Understanding the factors that influence oil extraction is essential for designing, optimizing, and controlling extraction processes. This can be achieved by conducting kinetic, diffusion, and thermodynamic studies. After extraction, the oil–solvent mixture is heated to evaporate and recover the solvent, leaving behind the crude oil. This crude oil may then undergo refining processes to remove impurities, improve physicochemical quality, and increase its commercial value and applicability across various industrial sectors.^10,11^

Despite the widespread application of oil extraction to various plant species, there is a lack of research focused on the oil extraction of *A. tessmannii* kernels and the physicochemical characteristics of the extracted oil. Hence, the present work aims to evaluate the chemical composition of *A. tessmannii* kernel, explore the parameters for lipid extraction, assess the kinetics and diffusivity coefficients of the extraction process, and characterize the obtained oil regarding its fatty acid profile and physical and chemical properties.

## 2 Material and Methods

### 2.1 Material Acquisition and Preparation

*A. tessmannii* ripened fruits were harvested directly from the soil by extractivists from December 2022 to February 2023. Figure 1 presents the location where the harvest was performed, at the Mogno State Forest, in Tarauacá, Acre, Brazil (8°08′08.0″S 70°45′54.0″W). After collection, the fruits were transferred to a local cooperative, *Cooperativa de Produtores Familiares e Economia Solidária da Floresta Estadual do Mogno* (COOPERMOGNO), where they were naturally dried and stored in a protected environment at 10 cm above the soil level. The parts of the fruit were separated by power and chainsaws, and the kernels were fragmented by maceration. Samples were kept at −18 °C until further experiments.

**Figure 1:**
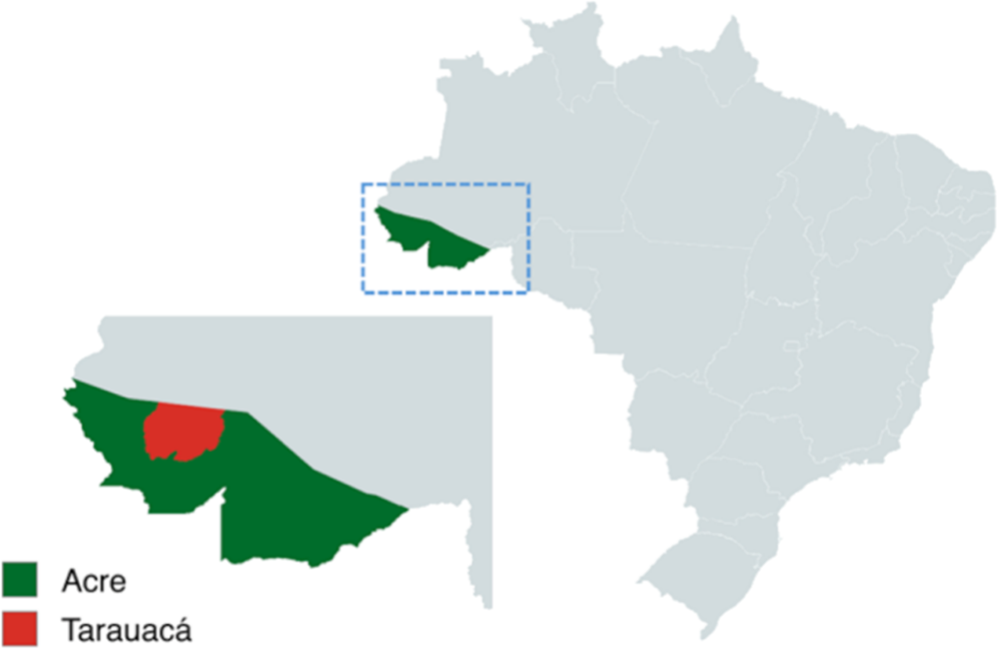
Harvest location of ripe *Attalea tessmannii* fruits: Mogno State Forest, Tarauacá, Acre, Brazil (8°08′08.0″S 70°45′54.0″W).

### 2.2 Kernel Characterization

The chemical proximal composition of kernels was determined according to AOAC methods.^12^ The moisture content was evaluated by drying at 105 °C overnight. The ash content was evaluated in a muffle at 550 °C for 6 h. Protein content was determined by Kjeldahl method using a value of 5.3 for the *N* factor. The total lipid content was assessed following the Bligh & Dyer methodology,^13^ with methanol and chloroform as solvents.^13^ After lipid quantification, samples were stored at −18 °C for further analyses. The carbohydrate content was determined by difference, which was calculated by subtracting the measured proportions of moisture, ash, protein, and lipids from the total composition (100%).

The total neutral lipid content of the kernels was determined using the Soxhlet apparatus with hexane as the solvent at the condensing temperature. For that, 2 g of sample was placed in a paper cartridge, and 150 mL of solvent was refluxed for 6 h. Then, the solvent underwent rotatory evaporation, and lipids were quantified through gravimetric analysis. Following quantification, samples were stored at −18 °C for subsequent analyses.

### 2.3 Lipid Extraction

Lipid extraction was performed using hexane (5:1 solvent-to-kernel, w/w ratio) at 25, 50, and 60 °C under agitation with a magnetic stirrer at 200 rpm. The glass flasks containing the samples were immersed in a water bath, and samples were withdrawn at 15, 30, 60, 120, 180, and 240 min. The total amount of lipids in the extracts was quantified gravimetrically after centrifugation and solvent evaporation at 70 °C.

### 2.4 Kinetic Analyses

The experimental data were fitted to the pseudo-first-order and Brimberg models, given by eqs 1 and 2, respectively. These models are commonly used to describe oil adsorption processes. Since solid–liquid extraction is the inverse of adsorption, adsorption kinetics equations can effectively be applied to model extraction data.^14,15^

1 $$\frac{m_{l}}{m_{tl}}=1-\exp\left(-k_{1}t\right)$$

2 $$\frac{m_{l}}{m_{tl}}=1-\exp\left(-k_{2}t^{n}\right)$$

in which *m*_l_ is the lipid content at time *t* (g 100 g^–1^), *m*_tl_ is the total lipid content of the kernel (g 100 g^–1^), *t* is the time (min), *k*_1_ and *k*_2_ are the extraction rate constants (min^–1^) and *n* is the model coefficient.

The temperature effect on the extraction rate constant was calculated by the Arrhenius equation, presented in eq 3.

3 $$k=k_{0}\exp\left(\frac{-E_{a}}{RT}\right)$$

in which *k* is the extraction rate constant (min^–1^), *k*_0_ is the frequency factor (min^–1^), *E*_a_ is the activation energy (J mol^–1^), *R* is the ideal gas constant (8.314 J mol^–1^ K^–1^), and *T* is the absolute temperature (K).

### 2.5 Diffusion Analysis

Diffusivity was determined using the modified Fick’s law of diffusion,^16^ assuming a homogeneous medium, spherical particles, and constant concentration, according to eq 4.

4 $$\frac{M_{t}}{M_{\infty }}=1-\sum_{n=1}^{\infty }A_{n}\;\exp\left(-B_{n}t\right)$$

where *t* is the time (s), *A*_*n*_ and *B*_*n*_ are the coefficients of the model that involve the diffusion coefficient, and *M*_*t*_ and *M*_∞_ are the masses of oil (kg of oil kg^–1^) that diffused in time *t* and infinite time, respectively. This model assumes that the solvent washes away the oil on the particle surfaces in a short period through a nondiffusive process. For sufficiently long times, Fick’s equation can be rewritten according to eq 5, where *A* is the pre-exponential coefficient, given by eq 6.

5 $$\frac{M_{t}}{M_{\infty }}=1-A\;\exp\left(-B_{1}t\right)$$

6 $$A=\left(1-\frac{M_{t}}{M_{\infty }}\right)A_{1}\;\exp\left(-B_{1}t_{0}\right)$$

The coefficients *A*_1_ and *B*_1_ can be obtained by eqs 7 and 8, respectively. The equation for determining *A*_1_ is associated with spherical geometry and *B*_1_ with the effective diffusivity coefficient, where *D*_*e*_ is the effective diffusion coefficient (m^2^ s^–1^) and *R* is the average radius of the particle (*m*).

7 $$A_{1}=\frac{6}{\pi^{2}}$$

8 $$B_{1}=\frac{D_{e}\times \pi^{2}}{R^{2}}$$

### 2.6 Oil Characterization

#### 2.6.1 Analysis of Fatty Acids Methyl Esters

The lipids (30 mg) were esterified according to Hartman et al.^42^ using 500 μL of KOH (0.1 M). The mixture was vortexed and kept in a water bath at 60 °C for 1.5 h. Then, 1.5 mL of 1 M H_2_SO_4_ was added to the flasks, and the mixture was incubated at 60 °C for 1.5 h. After cooling, the samples were vortexed with 2 mL of *n*-hexane, followed by a rest (10 min) to facilitate phase separation. The *n*-hexane phase was analyzed according to Borges et al.^17^ in a gas chromatograph (GC–FID, Shimadzu, 2010AF, Tokyo, Japan), equipped with a capillary column of fused silica SP-2560 (100 m × 0.25 mm x 0.2 μm) and using a flame ionization detector (FID). The carrier gas was N_2,_ and the flame gases were H_2_ and synthetic air. The sample split ratio was 1:100. The column temperature was adjusted to 100 °C for 15 min and then increased to 250 °C at a heating ramp of 4 °C min^–1^, remaining at this temperature for 45 min. The injector and detector temperatures were 250 and 255 °C, respectively. For fatty acid identification, the retention times were compared to those of a methyl ester standard (Supelco, 37-component FAME mix) previously analyzed by gas chromatography–mass spectrometry (GC–MS).

#### 2.6.2 Analysis of Iodine, Acidity, Peroxide, and Saponification Indices

The iodine, acidity, peroxide, and saponification indices were determined using a nuclear magnetic resonance (NMR) spectrometer (Bruker High Field, model 400 MHz Ascend, Rheinstetten, Germany) with a 9.4 T magnet (400 MHz at ^1^H) and a 5 mm diameter probe. For spectra acquisition, 20 mg of lipids were dissolved in 0.7 mL of CDCl_3_ and introduced into the equipment. A standard ^1^H pulse sequence was employed, consisting of a 90° pulse, an acquisition time of 9.109 s, 2000 scans, and a spectral window of 24.03 kHz.

#### 2.6.3 Fourier Transform Infrared Spectroscopy and Differential Scanning Calorimetry

The lipid extracts were analyzed by Fourier transform infrared (FTIR) spectroscopy (Shimadzu, Prestige 21, 210.045, Japan) in the range of 400–4000 cm^–1^. The thermal properties of the extracts were determined by differential scanning calorimetry (DSC) (Shimadzu, DSC-60, Japan).

### 2.7 Statistical Analysis

Experimental data were analyzed using analysis of variance (ANOVA) and Tukey’s test (95% confidence) with Statistica 13.5 (TIBCO Software Inc.). The kinetic and diffusion parameters were obtained by nonlinear regression using the MatLab software, employing the Levenberg–Marquardt algorithm. The quality of fit and accuracy were evaluated by the sum of square errors (SSE) (eq 9), the determination coefficient (*R* ^2^) (eq 10), the adjusted determination coefficient (*R*_adj_^2^) (eq 11), and the root-mean-square error (RMSE) (eq 12).

9 $$SSE=\sum_{i=1}^{n}{\left(y_{i,model}-y_{i,\exp}\right)}^{2}$$

10 $$R^{2}=\left(\frac{\sum_{i=1}^{n}{\left(y_{i,\exp}-{\mathrm{y̅}}_{i,\exp}\right)}^{2}-\sum_{i=1}^{n}{\left(y_{i,\exp}-{\mathrm{y̅}}_{i,model}\right)}^{2}}{\sum_{i=1}^{n}{\left(y_{i,\exp}-{\mathrm{y̅}}_{i,\exp}\right)}^{2}}\right)$$

11 $$R_{adj}^{2}=1-\left(1-R^{2}\right)\cdot \left(\frac{n-1}{n-p}\right)$$

12 $$RMSE=\sqrt{\frac{\sum_{i=1}^{N}{\left(y_{i,\exp}-y_{i,model}\right)}^{2}}{N}}$$

where *N* is the number of experimental points, *y*_*i*,model_ is each value of the *y* predicted by the fitted model, *y*_*i*,exp_ is each value of *y* measured experimentally, ${\mathrm{y̅}}_{i,\exp}$ is the average of *y* experimentally measured, ${\mathrm{y̅}}_{i,model}$ is the average of predicted values, and *p* is the number of parameters of the fitted model.

## 3 Results and Discussion

### 3.1 Chemical Composition of the Kernel

*A. tessmannii* kernel presented 64.19 ± 3.77% of total lipids, 22.53 ± 2.66% of proteins, 11.06 ± 1.57% of carbohydrates, 3.11 ± 0.24% of ash, and 2.04 ± 0.03% of moisture. High contents of oil were expected in the almond. Additionally, the presence of high protein content indicates the potential of this kernel as a nutritional resource. The neutral lipid content analysis, performed with hexane, resulted in 55.68 ± 2.44% lipids, indicating a low amount of polar lipids in the almonds. To the best of our knowledge, the proximate composition of the material studied in this work has not been previously documented in the literature. Similar lipid content (62%) was found for babassu (*A. speciosa* *M*.) kernel by Oliveira et al.^18^ The authors also reported 8% protein, 28% carbohydrates, and 1% ash. Moreover, the results obtained in the presented work for total lipids, protein, and ash content are within the range reported by Venkatachalam and Sathe^20^ for edible nuts: 42.88–66.71%, 7.5–21.56%, and 1.16–3.28%, respectively.

### 3.2 Extraction Kinetics and Activation Energy

Figure 2 presents the lipid content over time at all analyzed temperatures, with the lines representing the Brimberg model fitted to the experimental data. It is possible to observe two distinct stages during lipid extraction: an initial rapid phase, often referred to as the washing stage, followed by a slower phase driven by diffusion. This two-step behavior is common in solid–liquid extraction systems and results from the transition from easily accessible lipids on the particle surface to those found within the cellular structure, which require diffusion through the matrix. In the washing stage, the solvent penetrates the solid matrix, disrupting cell structures. As a result, internal compounds become exposed and are rapidly transferred into the extraction medium.^19^

**Figure 2:**
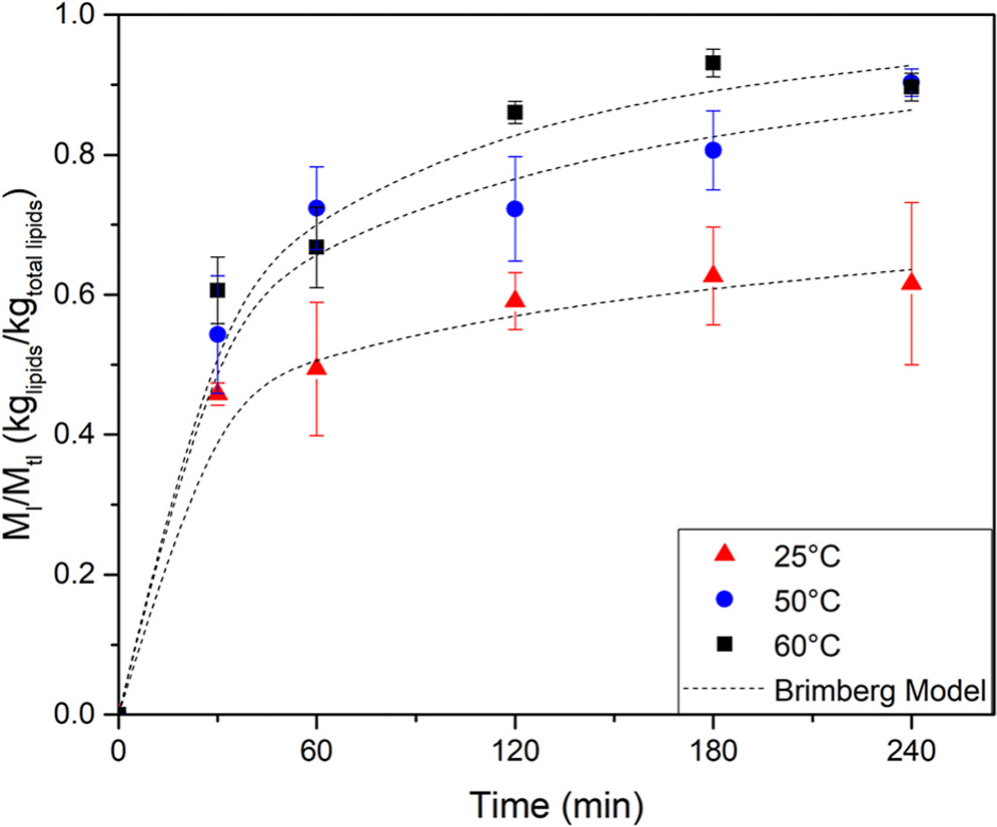
Lipid extraction kinetics from *Attalea tessmannii Burret* at different temperatures.

The results showed that lipid extraction increased with temperature, reaching its maximum at 60 °C after 180 min, yielding 51.84% of lipids. In contrast, at 25 °C, the extraction yield was approximately 1.5 times lower than the values obtained at 60 °C, with a maximum lipid content in the extracts of 32.9% achieved after 120 min. The increase in lipid yield with temperature is attributed to the reduction in solvent viscosity, as well as the enhanced solubility and diffusivity of lipids in the solvent, which facilitate the mass transfer.^21^ Similar results were found by Alale et al.^24^ that extracted oil from shea nut kernels using petroleum ether and *n*-hexane at 45–60 °C found that steady-state conditions occur between 110 and 130 min.

Table 1 presents the kinetic parameters and statistical indices obtained by fitting the experimental data to pseudo-first-order and Brimberg models. The Brimberg model was the most suitable for representing the extraction kinetics at all temperatures, as evidenced by the highest values of *R*_adj_^2^ and the lowest values of SSE and RMSE. The adequacy of the Brimberg model suggests that lipid extraction from *A. tessmannii* follows a nonlinear kinetic pattern with a variable extraction rate. The kinetic constant *k*_2_ decreased as the temperature increased, indicating that the extraction rate was higher at lower temperatures (25–50 °C) and decreased at 60 °C. In contrast, the parameter *n* increased with temperature, suggesting a tendency toward more linear kinetic behavior at higher temperatures. The variation in kinetic constants compared to other materials reflects differences in matrix structure and lipid accessibility. Pohndorf et al.^23^ evaluated the lipid extraction from *Spirulina* sp. at 20–60 °C, and the Brimberg model was also suitable to describe experimental data. These researchers obtained values of *k* from 0.113 to 0.446 min^–1^ and values of *n* from 0.43 to 0.75. Other authors evaluated the first-order model for the lipid extraction process from Ghana shea nut at 20–35 °C and obtained values of *k* from 0.0076 to 0.0118 min^–1^.^24^

**Table 1:** Kinetic Parameters of Lipid Extraction from *Attalea tessmannii Burret* at 25, 50, and 60 °C

	25 °C	50 °C	60 °C
	Pseudo-First Order		
*k*_1_ (min^–1^)	0.00703 ± 0.001^b^	0.01782 ± 0.002^a^	0.02197 ± 0.002^a^
*R* ^2^	0.4947	0.8538	0.9405
SSE	0.1425	0.07697	0.03617
RMSE	0.1688	0,1241	0,08505
	Brimberg Model		
*k*_2_ (min^–1^)	0.2614 ± 0.05^a^	0.1945 ± 0.03^ab^	0.1453 ± 0.05^b^
*N*	0.2468 ± 0.08^b^	0.4247 ± 0.06^a^	0.528 ± 0.06^a^
*R* ^2^	0.9952	0.9849	0.9906
*R*_adj_^2^	0.994	0.984	0.9883
SSE	0.00134	0.00793	0.528
RMSE	0.01833	0.04451	0.03774

Figure 3 shows the Arrhenius plot used to determine the activation energy of the lipid extraction process. The activation energy of the lipid extraction was 27.5 kJ mol^–1^. Activation energy is the minimum energy required to begin an extraction process, and lower activation energy values would indicate a predominantly washing-controlled process, whereas higher values could indicate resistance to mass transfer.^25^ The moderate activation energy value obtained in the present work indicates that the process is mainly diffusion-controlled, which aligns with the nature of hexane extraction and the structural characteristics of oil-rich kernels. This result is similar to the ones obtained by Shuai et al.^22^ (26.42–29.59 kJ mol^–1^) for macadamia oil extraction. Moreover, Zhang et al.^26^ calculated the activation energy using different solvents for the extraction of *Pachira macrocarpa* seeds oil and obtained values ranging from 25.71 to 32.35 kJ mol^–1^.

**Figure 3:**
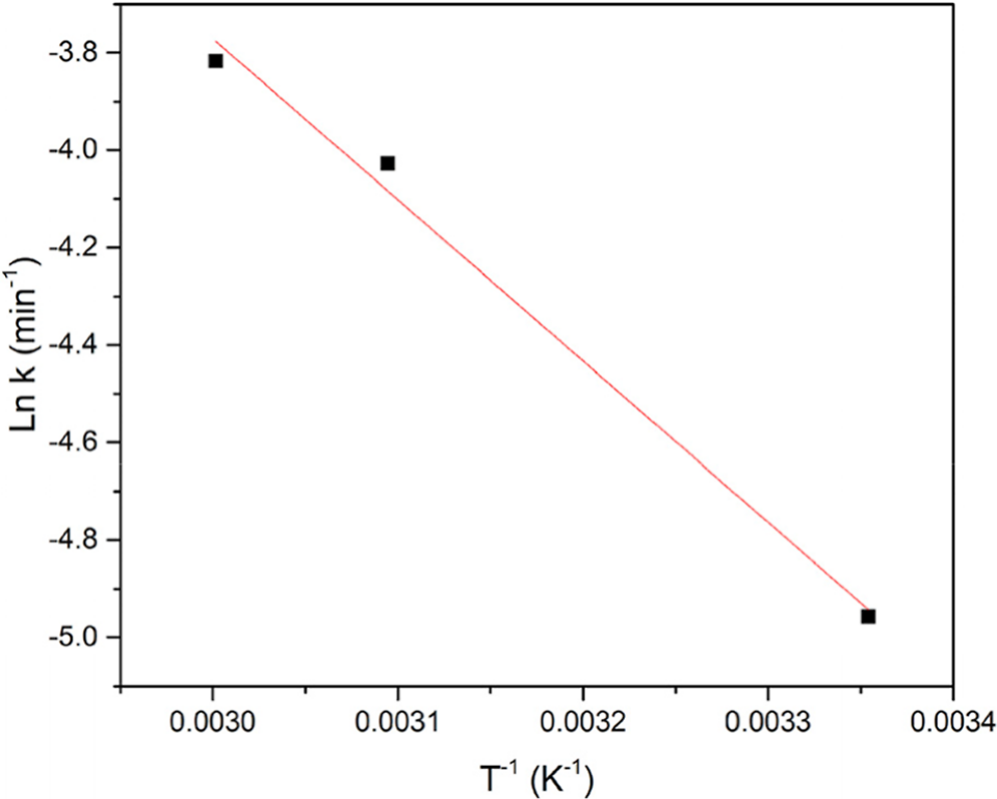
Arrhenius plot for lipid extraction from *Attalea tessmannii* kernel.

### 3.3 Diffusion Analyses

Table 2 presents the results of fitting the diffusive model to the experimental data, along with the statistical parameters of the fit. The values of the A coefficient decreased with the increase in temperature. The decrease in the A coefficient with temperature may reflect a reduction in the contribution of external mass transfer in comparison to internal diffusion, as higher temperatures tend to enhance solvent penetration. Moreover, an increase in the extraction rate was observed in the first minutes of the operation, as shown in Figure 2, indicating that this initial stage was relatively small and corresponds to the washing of the oil from the surface of the sample. Therefore, diffusion dominated the extraction process over time, supporting the applicability of the diffusive model. Furthermore, the increase in the B coefficient with temperature reinforces the dominance of the diffusion-controlled regime, in which the internal mass transfer becomes the limiting step.^21^ At higher temperatures, especially 60 °C, the model fit improved, as evidenced by an adjusted coefficient of determination (*R*_adj_^2^) of 0.95 and a lower root-mean-square error (RMSE).

**Table 2:** Adjustment Parameters of the Diffusion Model at Different Oil Extraction Temperatures from *Attalea tessmannii* Kernel

extraction temperature (°C)	25	50	60
*A* (×10^2^)	99.9	94.4	95.4
*B* (×10^4^)	1.18	2.71	3.78
SSE	0.0113	0.0091	0.0048
*R* ^2^	0.810	0.903	0.954
*R*_adj._^2^	0.783	0.889	0.947
RMSE	0.1062	0.0952	0.0693

The determination of the diffusivity coefficient (*D*_e_) is important for understanding the oil extraction phenomenon and simulating the behavior of industrial extractors. The diffusion coefficient increased from 1.8 × 10^–11^ to 5.8 × 10^–11^ m^2^/s with an increase in temperature from 25 to 60 °C, as shown in Figure 4. This increase in the diffusion coefficient with temperature can be attributed to the enhanced solubility of the crude oil and the solvent at higher temperatures, favoring solute diffusion.

**Figure 4:**
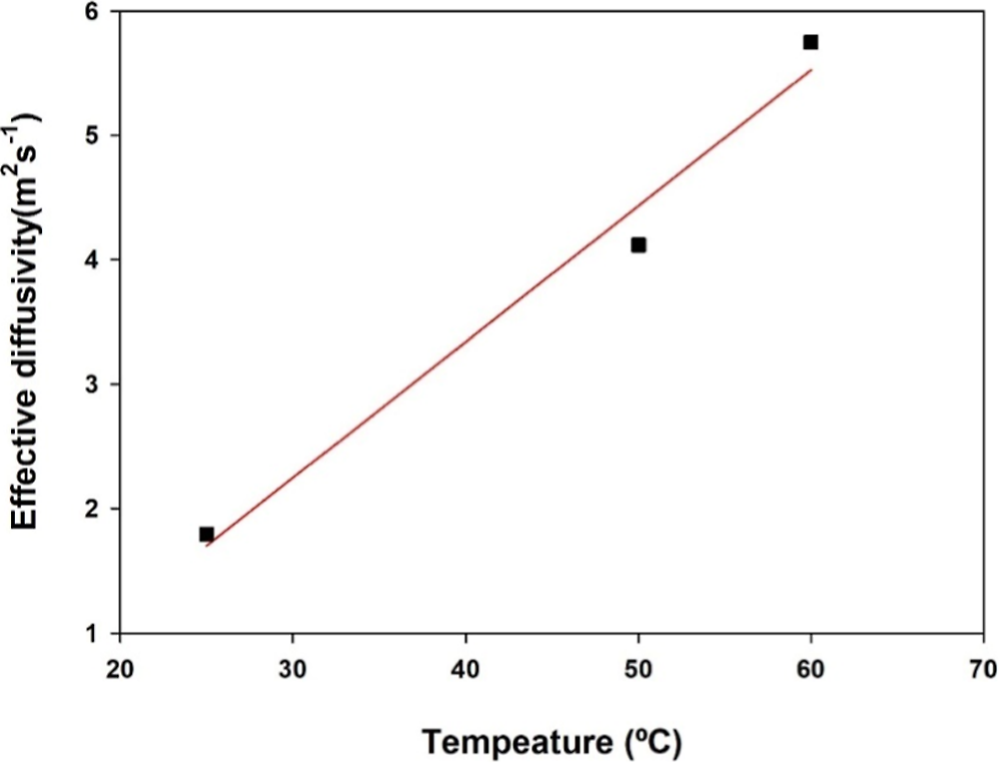
Influence of extraction temperature on effective diffusivity of oil from *Attalea tessmannii* kernel.

Crude oil extracted from oilseeds has some minor components. Among them are tocopherols, which are intrinsically linked to oil and phospholipids that are part of the cell membrane and are responsible for maintaining cellular integrity. The amount of these compounds in the material structure can affect the extraction rate and consequently the diffusion coefficient.^27^ Wickramasinghe Mudiyanselage and Wickramasinghe^28^ studied the extraction of canola oil with hexane as solvent, finding values for the diffusivity coefficient in the range of 1.3 × 10^–12^ to 3.0 × 10^–12^ m^2^/s, at temperatures from 25 to 60 °C, respectively. When compared to the extraction of wild coconut seeds, the difference in diffusion coefficient values can be attributed to the particle size and structural differences between the raw materials. Therefore, the morphological characteristics of *A. tessmannii* may contribute to the relatively high *D*_e_ values observed, which is relevant for the design of scalable extraction systems.

### 3.4 Oil Characterization

#### 3.4.1 Fatty Acids Methyl Esters (FAMEs) Profile

Table 3 shows the FAME profile of the oil extracted from *A. tessmannii* kernels at different temperatures. The results showed that the FAME profile did not vary with the increase in temperature, indicating that temperature had no significant impact on the fatty acid composition. This stability can be attributed to the high content of saturated FAMEs in the oil, which comprises around 90% of its composition. This thermal stability suggests that the extraction conditions (25–60 °C) preserved the fatty acids, preventing degradation, isomerization, or oxidation processes commonly associated with higher temperatures. The analysis revealed a composition rich in lauric acid (C12:0), accounting for approximately 50% of the oil. This fatty acid is a primary source of medium-chain triglycerides, which are rapidly metabolized to provide energy and are widely used in infant formulas and athletic supplements. Additionally, the oil contained nearly 14% myristic acid (C14:0), and 8% oleic acid (C18:1). This fatty acid profile is comparable to that reported for babassu kernel, palm kernel, and coconut oil.^5,28,29^ According to Neto et al.,^5^ the fatty acid methyl ester profile of babassu kernel oil includes 40–55% of C12:0, 11–27% of C14:0, 7.8–20% of C18:1, 5.2–11% of C16:0, 2.6–7.3% of C8:0, 1.8–7.4% of C18:0, 1.4–6.6% of C18:2. Similarly, palm kernel oil contains 48% of lauric acid (C12:0), 16% of myristic acid (C14:0), 15% of oleic acid, and 8% of palmitic acid.^28^ The similarity in fatty acid composition between *A. tessmannii* and other palm kernels reinforces the potential application of this species as a source of medium-chain fatty acids for the food, pharmaceutical, and biofuel industries, and its compositional stability across different extraction temperatures may offer industrial advantages.

**Table 3:** Fatty Acid Methyl Ester Profile of the Lipid Extracts from *Attalea tessmannii* Kernel Obtained with Hexane at 25, 50, and 60 °C

fatty acid methyl ester (area, %)	25 °C	50 °C	60 °C
caproic acid (C6:0)	0.28 ± 0.07^a^	0.38 ± 0.03^a^	0.35 ± 0.03^a^
caprylic acid (C8:0)	7.14 ± 0.81^a^	8.05 ± 0.63^a^	8.19 ± 0.62^a^
capric acid (C10:0)	6.78 ± 0.21^a^	7.12 ± 0.13^a^	6.97 ± 0.25^a^
undecylic acid (C11:0)	0.03 ± 0.00^a^	0.03 ± 0.00^a^	0.03 ± 0.00^a^
lauric acid (C12:0)	49.65 ± 1.5^a^	50.07 ± 4.0^a^	49.60 ± 2.1^a^
tridecylic acid (C13:0)	0.03 ± 0.00^a^	0.04 ± 0.00^a^	0.04 ± 0.00^a^
myristic acid (C14:0)	14.61 ± 0.82^a^	14.16 ± 1.35^a^	14.28 ± 0.52^a^
palmitic acid (C16:0)	7.42 ± 0.23^a^	6.98 ± 0.99^a^	7.12 ± 0.78^a^
stearic acid (C18:0)	3.18 ± 0.53^a^	2.95 ± 0.76^a^	3.04 ± 0.41^a^
oleic acid (C18:1)	8.62 ± 0.91^a^	8.04 ± 1.02^a^	8.24 ± 0.54^a^
linoleic acid (C18:2)	2.10 ± 0.13^a^	1.92 ± 0.17^a^	2.02 ± 0.09^a^
arachidic acid (C20:0)	0.06 ± 0.01^a^	0.06 ± 0.00^a^	0.05 ± 0.00^a^
eicosenoic acid (C20:1)	0.04 ± 0.00^a^	0.04 ± 0.00^a^	0.04 ± 0.00^a^
heneicosanoic acid (C21:0)	0.00 ± 0.00^a^	0.07 ± 0.00^a^	0.00 ± 0.00^a^
behenic acid (C22:0)	0.02 ± 0.00^a^	0.03 ± 0.00^a^	0.00 ± 0.00^a^
Lignoceric acid (C24:0)	0.05 ± 0.00^a^	0.07 ± 0.01^a^	0.03 ± 0.00^a^
**total saturated fatty acids (SFAs)**	**89.24**	**90.00**	**89.70**
**total monounsaturated fatty acids (MUFAs)**	**8.65**	**8.07**	**8.28**
**total polyunsaturated fatty acids (PUFAs)**	**2.10**	**1.92**	**2.02**

#### 3.4.2 Peroxide, Saponification, Free Fatty Acid and Iodine Indices

Table 4 presents the physicochemical parameters of the oil extracted at 25, 50, and 60 °C. In general, the physicochemical parameters obtained in this study did not vary with temperature and were consistent with those reported by other authors for oils containing high levels of saturated fatty acids, such as coconut and babassu oils.^7,30^ This thermal stability can be attributed to the chemical structure of saturated fatty acids, which are less reactive and more resistant to thermal degradation than unsaturated ones. Therefore, variations in extraction temperature within the studied range (25–60 °C) are unlikely to affect the physicochemical integrity of oils rich in these compounds.

**Table 4:** Physicochemical Characterization of the *Attalea tessmannii* Kernel Oil Obtained at 25, 50, and 60 °C

parameters	25 °C	50 °C	60 °C
peroxide value (PV) (mEq_peroxides_ kg_oil_^–1^)	0.08 ± 0.01^a^	0.09 ± 0.00^a^	0.10 ± 0.00^a^
saponification value (SV) (mg KOH g^–1^)	240.08 ± 1.41^a^	240.23 ± 1.79^a^	242.96 ± 1.90^a^
free fatty acid (FFA) (%, oleic acid)	2.87 ± 0.05^a^	2.82 ± 0.06^a^	2.76 ± 0.09^a^
iodine value (IV) (cgI_2_ g^–1^)	6.74 ± 0.81^a^	7.31 ± 0.99^a^	8.71 ± 0.72^a^

Moreover, the values presented in Table 4 are in the expected range for biodiesel production,^31^ and are within the limits required by the regulations of Codex for vegetable oils.^32^ Furthermore, due to the similarity of *A. tessmannii* kernel oil and babassu oil, this oil may also be interesting for cosmetic use, due to the emollient properties of saturated fatty acids and anti-inflammatory activity of lauric, oleic, and myristic acids.^7,18^ These fatty acids penetrate the skin barrier effectively and support skin hydration and protection. Lauric acid, in particular, possesses antimicrobial and anti-inflammatory properties, making it beneficial for formulations aimed at sensitive or acne-prone skin.^33^

The low peroxide values (PV) indicated low levels of free radicals and lipid oxidation. These values are also in agreement with the low levels of free fatty acids (FFA), suggesting that *A. tessmannii* kernel oil presents high quality and stability. Similarly, Pandiselvam et al.^34^ reported PV values of zero and FFA levels varying from 0.07 to 0.71 for coconut oil. Regarding the saponification values (SV), the result obtained in the present work was similar to the one obtained for coconut (244.19 mg KOH g^–1^)^30^ and for babassu oil (249 mg KOH g^–1^).^7^ The SV is an indication of the average molecular weight of fatty acids in the oil, corroborating the results found for the FAME profile, which indicated the presence of short-chain fatty acids. The iodine value (IV) measures the level of unsaturation of oils. This parameter is important for biodiesel production, as the higher this value, the lower the oil oxidative stability. Similar values (6.3–9.4 cgI_2_ g^–1^) were obtained for coconut oil by Pandiselvam et al.^34^

#### 3.4.3 Fourier Transform Infrared Spectroscopy and Differential Scanning Calorimetry

Figure 5 presents the FTIR spectra of *A. tessmannii* kernel oil. The spectra are consistent with those of other vegetable oils, and the main peaks were observed in the regions of 1100–1250 cm^–1^, 1700–1800 cm^–1^, and 2800–3100 cm^–1^.^35^ The peaks within the 1100–1250 cm^–1^ range correspond to −C–O stretching and −CH_2_– bending vibrations. In the 1700–1800 cm^–1^ region, the peaks are attributed to C=O stretching, while the 2800–3100 cm^–1^ range is associated with −C–H (−CH_2_) stretch vibrations.^36–38^ These characteristic peaks are typically associated with triglyceride structures found in vegetable oils, confirming the presence of ester functional groups. The strong band around 1745 cm^–1^ (C=O) is indicative of ester carbonyl stretching, while bands near 2920 and 2850 cm^–1^ correspond to asymmetric and symmetric stretching of CH_2_ groups in long-chain fatty acids. The spectrum, therefore, corroborates the lipid nature and high degree of saturation of the sample.

**Figure 5:**
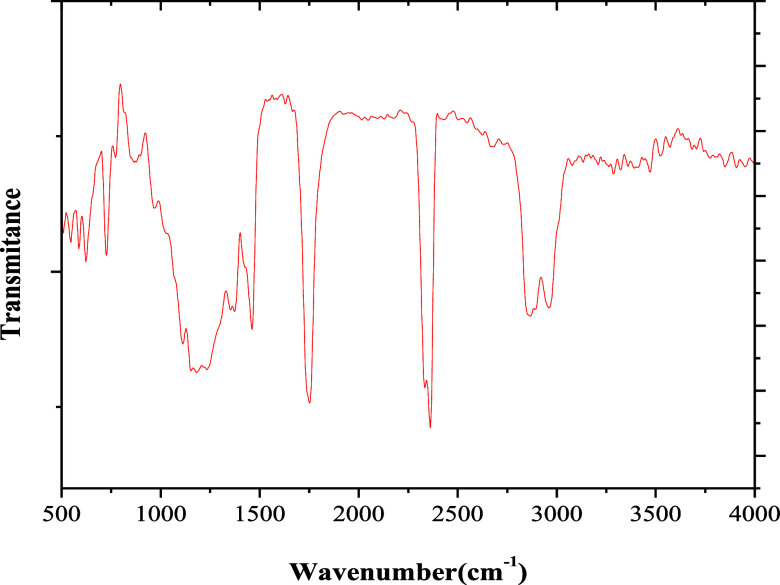
*Attalea tessmannii* kernel oil FTIR spectra.

The results of the DSC analyses of the *A. tessmannii* kernel oil are presented in Figure 6. The findings indicate that the melting point of the oil ranges from 0 to 36 °C, with a transition peak at approximately 26 °C. Similar results were obtained by Bauer et al. (2020),^7^ that found a melting point in the same value for babassu oil. Tan and Man^39^ also found melting points of approximately 26 °C for palm oil and 22 °C for coconut oil. The melting behavior reflects the high concentration of medium-chain saturated fatty acids, which contributes to the semisolid consistency of the oil at room temperature. This thermal profile is favorable for cosmetic and food applications that require fats with good spreadability and stability at ambient conditions.^40^ Furthermore, the relatively low melting point is also beneficial for biodiesel applications, as it suggests favorable handling and storage characteristics, reducing or eliminating the need for preheating prior to processing or use.^41^

**Figure 6:**
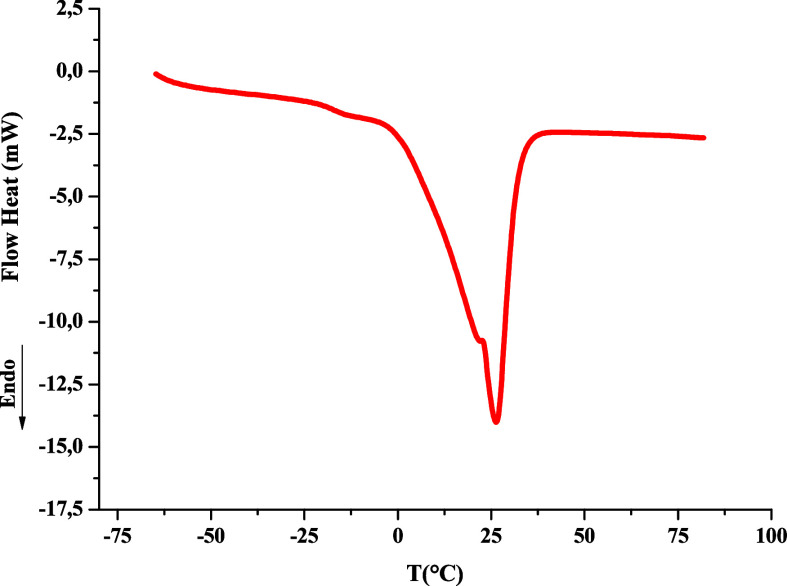
Thermogram of the crystallization and melting of *Attalea tessmannii* kernel oil.

With DSC, an enthalpy change of −79.68 J g^–1^ was obtained during the phase transition. This high enthalpy of fusion is characteristic of oils rich in saturated fats, which have strong van der Waals interactions due to the linear structure of their fatty acid chains. Figure 7 displays the solid fat content (SFC) curves for the *A. tessmannii* oil. The sample exhibited an SFC of 45% at 22 °C and 35% at 25 °C. At 10 °C, only 15% of the sample was liquefied, emphasizing the characteristics of an oil that is predominantly composed of saturated fatty acids. The relatively high SFC at room temperature reinforces the potential of this oil in energy, food, and cosmetic applications, as oils with elevated SFC values tend to exhibit better oxidative stability and longer shelf life.

**Figure 7:**
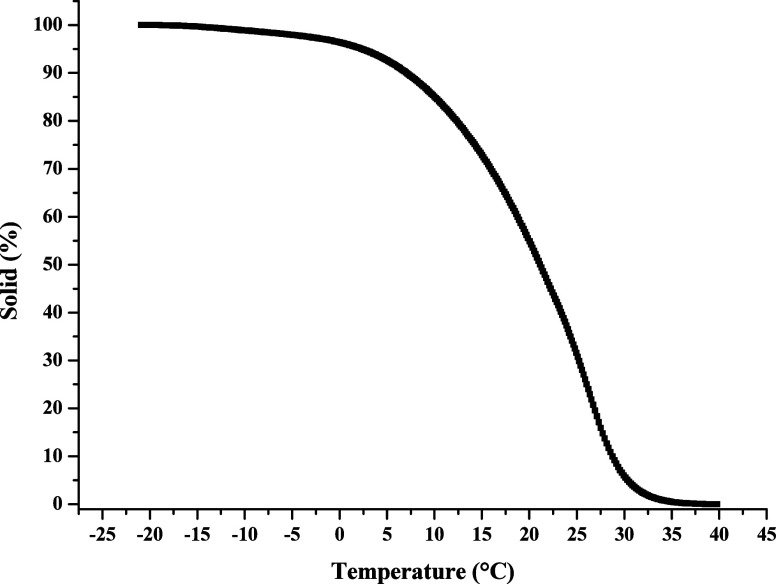
TG/DTG curves of *Attalea tessmannii* kernel oil.

## 4 Conclusion

The present study evaluated the proximate composition of *A. tessmannii* kernel, investigated the oil extraction process through kinetic and diffusivity analyses, and carried out the physicochemical characterization of the extracted oil. The kernel presented high lipid content (64.19%), and the Brimberg model was the most suitable for describing the extraction process at all temperatures (25–60 °C). The diffusion coefficient increased with temperature due to the enhanced solubility of the crude oil at higher temperatures. The oil, obtained at 25–60 °C, contained approximately 90% short-chain fatty acids, primarily lauric acid. The physicochemical properties of the oil remained stable across a range of temperatures, and, due to its high thermal and oxidative stability, it falls within the expected range for biodiesel production and meets Codex standards for vegetable oils. Additionally, *A. tessmannii* oil shows potential for cosmetic applications, particularly due to its emollient properties, attributed to saturated fatty acids. These results show that *A. tessmannii* has economic potential and can be a sustainable resource for both traditional Amazonian communities and industry. Its use can help support local economies and contribute to rainforest conservation.
